# Low failure rates and satisfactory PROMs in ACL reconstruction with decellularized porcine xenograft: Short‐term results in middle‐aged adults

**DOI:** 10.1002/jeo2.70712

**Published:** 2026-06-16

**Authors:** Gian Andrea Lucidi, Francesco Aparo, Nicolò Maitan, Giuseppe Gianluca Costa, Simone Natali, Daniele Screpis, Claudio Zorzi, Arcangelo Russo, Stefano Zaffagnini

**Affiliations:** ^1^ Clinica Ortopedica e Traumatologica II IRCCS Istituto Ortopedico Rizzoli Bologna Italy; ^2^ Università di Bologna (UniBo) Bologna Italy; ^3^ Università degli studi ‘Kore’ di Enna Enna Italy; ^4^ IRCCS Ospedale Sacro Cuore – Don Calabria Negrar di Valpolicella Italy

**Keywords:** allograft, anterior cruciate ligament, graft, pivot‐shift, xenograft

## Abstract

**Purpose:**

To evaluate the short‐term outcomes of arthroscopic anterior cruciate ligament (ACL) reconstruction (ACLR) using a decellularized porcine xenograft, focusing on patient‐reported outcome measures (PROMs), complications and failure rates.

**Methods:**

This retrospective study included 48 patients who underwent primary or revision ACLR with the Orthopure® xenograft. Preoperative and post‐operative International Knee Documentation Committee (IKDC) and Tegner scores were collected and showed remarkable improvements at different follow‐ups. Complications, reinterventions and failure rates were assessed. Post‐operative evaluations were conducted at 6, 12 and 24 months of follow‐up. The percentage of patients who achieved the patient acceptable symptom state (PASS) was calculated too.

**Results:**

The mean age at surgery was 39.9 ± 8.5 years, with a mean follow‐up of 19.5 ± 7.0 months. Primary ACLR was performed in 42 patients (87.5%), and revision surgery in 6 patients (12.5%). An associated meniscal procedure was performed in 38 cases (79.2%). Reoperation was required in 4 patients (8.1%) due to tibial tunnel enlargement or hardware‐related complications. Graft failure occurred in 2 patients (4.1%). PASS was achieved in 45 patients (94%).

**Conclusion:**

ACLR using a porcine‐derived xenograft appears to be a safe procedure with good clinical outcomes, a low rate of complications and graft failure. However, further studies with longer follow‐up and including high‐risk patients are warranted to better assess the long‐term performance of this graft.

**Level of Evidence:**

Level IV, case‐series.

**Abbreviations**
ACL: anterior cruciate ligament
ACLR: anterior cruciate ligament reconstruction
AM: anteromedial
CE: Conformité Européenne
FU: follow‐up
ICRS: International Cartilage Repair Society
IKDC: International Knee Documentation Committee
MCL: medial collateral ligament
MRI: magnetic resonance imaging
OA: osteoarthritis
PASS: patient acceptable symptom state
PCL: posterior cruciate ligament
PLC: posterolateral corner
SD: standard deviation
TAS: Tegner Activity Rating Scale
XT: xenograft tendon

## INTRODUCTION

Several graft options are available for anterior cruciate ligament (ACL) reconstruction (ACLR). Autografts, such as hamstring, quadriceps or patellar tendon grafts, are generally considered the gold standard due to their favourable integration and biomechanical properties [12]. However, allografts are also widely accepted, particularly in older patients with lower physical demands or in revision ACL procedures, where autograft options may be limited or less desirable [2, 7]. Despite their clinical utility, allografts present several limitations, including higher cost, limited availability, delayed biological incorporation and a potential—albeit low—risk of disease transmission. In an effort to overcome these drawbacks, alternative graft sources have been explored, including synthetic ligaments and xenografts.

Synthetic grafts, such as Ligament Augmentation and Reconstruction System (LARS®) [19], although initially promising, have demonstrated high failure rates, as well as complications such as synovitis, mechanical degradation and the release of intra‐articular debris, which have significantly limited their use in modern practice for ACLR [1, 9]. Xenografts, biological grafts derived from non‐human species, have also been investigated as a potential solution [21], especially in the setting of clinical trials on animal models [18]. Among them, decellularized porcine grafts have attracted interest due to their structural similarity to human tissue and immunological safety with modern processing systems [4, 6, 10, 18]. Nevertheless, previous studies on xenografts have yielded inconsistent results, with variability in graft incorporation, immune response and clinical outcomes [20].

OrthoPure® XT is a CE‐certified decellularized porcine‐derived tendon that has already been used in preclinical and clinical studies [13, 14], demonstrating promising results in terms of safety, integration and clinical outcomes, proving to be a valid alternative to autografts and allografts. However, there is still a need to provide real‐world evidence, in the form of clinical data, that can demonstrate the safety and performance of these xenogeneic grafts in ACLR. Based on these premises, the aim of our study was to conduct a multicentric evaluation of patients undergoing ACLR surgery using OrthoPure® XT, according to clinical outcomes as well as reoperations and failure rates.

## METHODS

Forty‐eight patients underwent ACLR (either primary or revision) with OrthoPure® XT in three Italian healthcare institutions. All surgical procedures were performed by experienced knee surgeons, who are all included among the authors. The study protocol was approved by the Institutional Review Board of Istituto Ortopedico Rizzoli (Protocol no. 0012799). All patients signed the informed consent form prior to surgery.

Patients were screened for inclusion in the study according to the following criteria:

### Inclusion criteria

- Age between 18 and 65 years at the time of ACL revision surgery;
- Age between 35 and 65 years at the time of primary ACLR surgery; and
- Male and female genders.

### Exclusion criteria

- Infectious, haematological, rheumatic or haemocoagulative pathology at the time of evaluation;
- Previous allergies, hypersensitivity reactions or religious constraints to the use of porcine‐derived material;
- Pregnant and/or breastfeeding status;
- Patients with open physis at the time of surgery; and
- Patients candidates for combined ACLR and meniscus transplant, osteotomy or cartilage procedures.

### Patient evaluation

Demographics and surgical details, such as patient age, sex and concurrent meniscal procedures, were obtained by chart review. Patients were evaluated pre‐operatively at several follow‐up (FU) visits, which were performed at 6, 12 and 24 months post‐operatively. The patients' outcomes were assessed using the Lysholm Knee score, Tegner Activity Rating Scale (TAS) and International Knee Documentation Committee (IKDC) score, which were collected both pre‐ and post‐operatively. Patients were also questioned whether they had undergone any additional surgeries on the index knee during the follow‐up period. Based on clinical evaluations, in cases of positive Lachman or anterior drawer tests or if the patient reported subjective knee instability, an MRI was prescribed to evaluate the graft status. Surgical failures were defined as the need for ACL graft revision or the evidence on MRI of an ACL graft tear.

### Surgical technique

**Figure 1:**
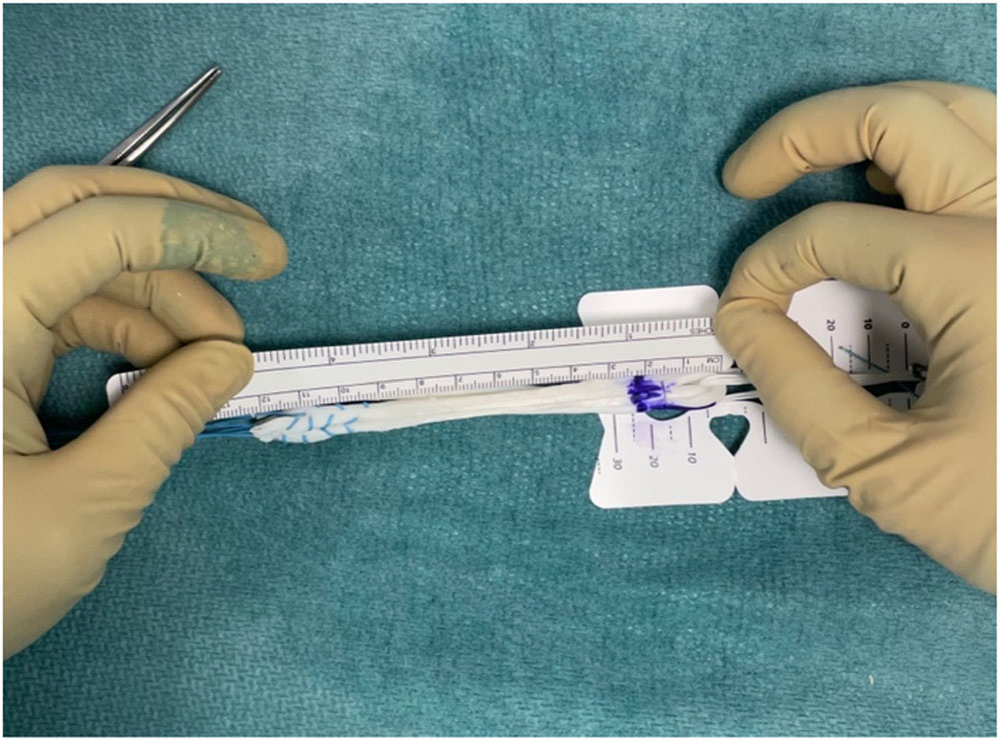
Xenograft preparation.

After clinical examination under anaesthesia, knee arthroscopy was first performed in all patients through standard anteromedial and anterolateral portals. Meniscal tears were addressed before proceeding with ACL reconstruction, either with repair or meniscectomy. All patients underwent ACL reconstruction with a porcine‐derived xenograft. OrthoPure® XT is a CE‐certified porcine‐derived tendon treated with a decellularization process which eliminates all native cells and immunogenic components, thus preserving biomechanical and histological characteristics of the graft. The xenografts are already pre‐prepared (Figure 1), and the average size of the graft was 8 mm.

The femoral tunnel was created aiming at the femoral footprint of the ACL with the knee hyperflexed through the anteromedial portal (AM) or with the knee flexed at 80–90° through the transtibial tunnel technique. The tibial tunnel was created by over‐reaming a guide pin directed from the medial aspect of the tibia to the tibial anatomical ACL footprint. The xenograft tendons were passed through the tibial tunnel, into the joint and into the femoral tunnel. The grafts were fixed at the femoral level using a rigid‐loop adjustable suspension mechanism, and at the tibial level with either bioabsorbable or non‐absorbable interference screws. In 12 patients, all treated at one centre, the tibial tunnel was created before the femoral tunnel using a modified transtibial technique [15].

### Rehabilitation protocol

Patients' rehabilitation protocol consisted of 1 month with progressive weight bearing on the operated limb with the aid of two crutches, followed by 2 additional weeks with the use of one crutch contralateral to the operated side. Physiotherapy‐assisted exercise for range of motion (ROM) recovery was started on post‐operative Day 3 in the absence of an associated meniscal suture or if a meniscectomy was performed. On the other hand, in the case of meniscal sutures, for the first post‐operative month, patients were advised to use reduced loading (from 10% to 50% depending on the meniscus lesion) on the operated knee and ROM recovery was started after 10 days. A knee brace was recommended for 4 weeks only in the case of meniscus suture. After complete wound healing (15–18 days), patients were advised to begin functional recovery with hydrotherapy. Patients were generally allowed to return to sports after 7–8 months from surgery. The rehabilitation protocol was standardized across the different centres involved in the study.

### Statistical analysis

Continuous variables were expressed as median with interquartile range (IQR). Normality of data distribution was assessed using the Shapiro–Wilk test. As the assumption of normality was not met, the Friedman test was applied to assess differences in outcome scores across the four time points. Post hoc pairwise comparisons were conducted using the Wilcoxon signed‐rank test with Bonferroni correction. All analyses were performed using MedCalc (MedCalc software, Acacialaan 23.3.7, Ostend, Belgium), and a *p* value < 0.05 was considered statistically significant.

## RESULTS

### Patients characteristics

After the application of inclusion and exclusion criteria, 48 patients were included in the study. The majority of patients were males (58.3%), and the average age at surgery was 39.9 ± 8.5 years old (age range = 23–64). The average follow‐up at final evaluation was 19.5 ± 6.97 months (range = 6.2–33.1).

Patients included in the study underwent primary ACLR in 42 (87.5%) cases, while revision ACLR was performed in 6 cases (12.5%). Moreover, intraoperative patient assessment showed that 34/48 (70.83%) patients had a medial meniscus lesion while 4/48 (8.33%) had a lateral meniscus lesion. Medial meniscus lesions were treated with repair (sutures) in 15 cases, meniscectomy in 9 cases and partial meniscectomy in 10 cases. Lateral meniscal lesions were managed with either repair (3 cases) or meniscectomy (1 case). In addition, eight (16.7%) patients had a chondral lesion, and six (12.5%) cases showed associated ligamentous lesions in addition to the ACL (Table 1). In addition, chondral lesions (ICRS grade 1–2), which were treated with minimally invasive procedures (e.g, debridement), were detected in eight patients (16.7%); specifically, these involved the lateral femoral condyle in two cases (25%) and the medial femoral condyle in six cases (75%). Associated procedures were performed in six patients, including two lateral extra‐articular tenodesis, two Hughston procedures, one combined posterior cruciate ligament (PCL) and medial collateral ligament (MCL) reconstruction and one posterolateral corner (PLC) reconstruction (Table 1).

**Table 1:** Demographic and surgical characteristics of the patients included in the study.

Total patients	48
Age at surgery (years)	39.9 ± 8.5
Mean follow‐up (months)	19.5 ± 6.97
Sex (males/females)	28 (58.33%)/20 (41.67%)
Medial meniscus lesion	34 (70.83%)
Repair	15
Meniscectomy	9
Partial meniscectomy	10
Lateral meniscus lesion	4 (8.33%)
Repair	3
Chondral lesion	8 (16.7%)
Medial femoral condyle	6
Lateral femoral condyle	2
Associated ligamentous lesion	6 (12.5%)
Lateral extra‐articular tenodesis	2
Hughston	2
PCL + MCL reconstruction	1
PLC reconstruction	1

Abbreviations: MCL, medial collateral ligament; PCL, posterior cruciate ligament; PLC, posterolateral corner.

### Patient‐reported outcome measures (PROMs)

**Figure 2:**
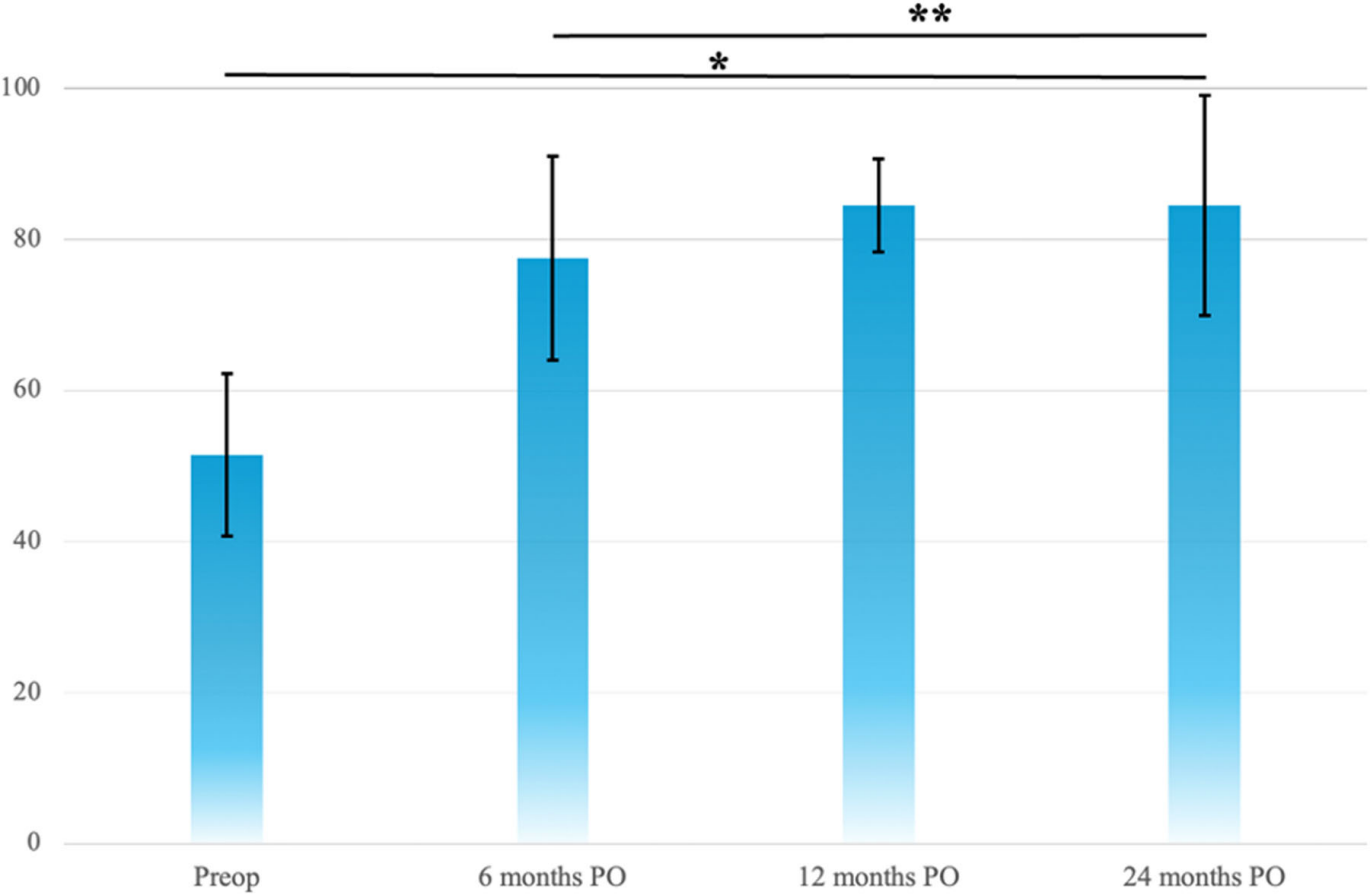
Mean IKDC scores. Vertical lines inside bars represent standard deviations, while horizontal lines with asterisks refer to statistically significant values (*p* < 0.05) across the follow‐up visits. IKDC, International Knee Documentation Committee.

**Figure 3:**
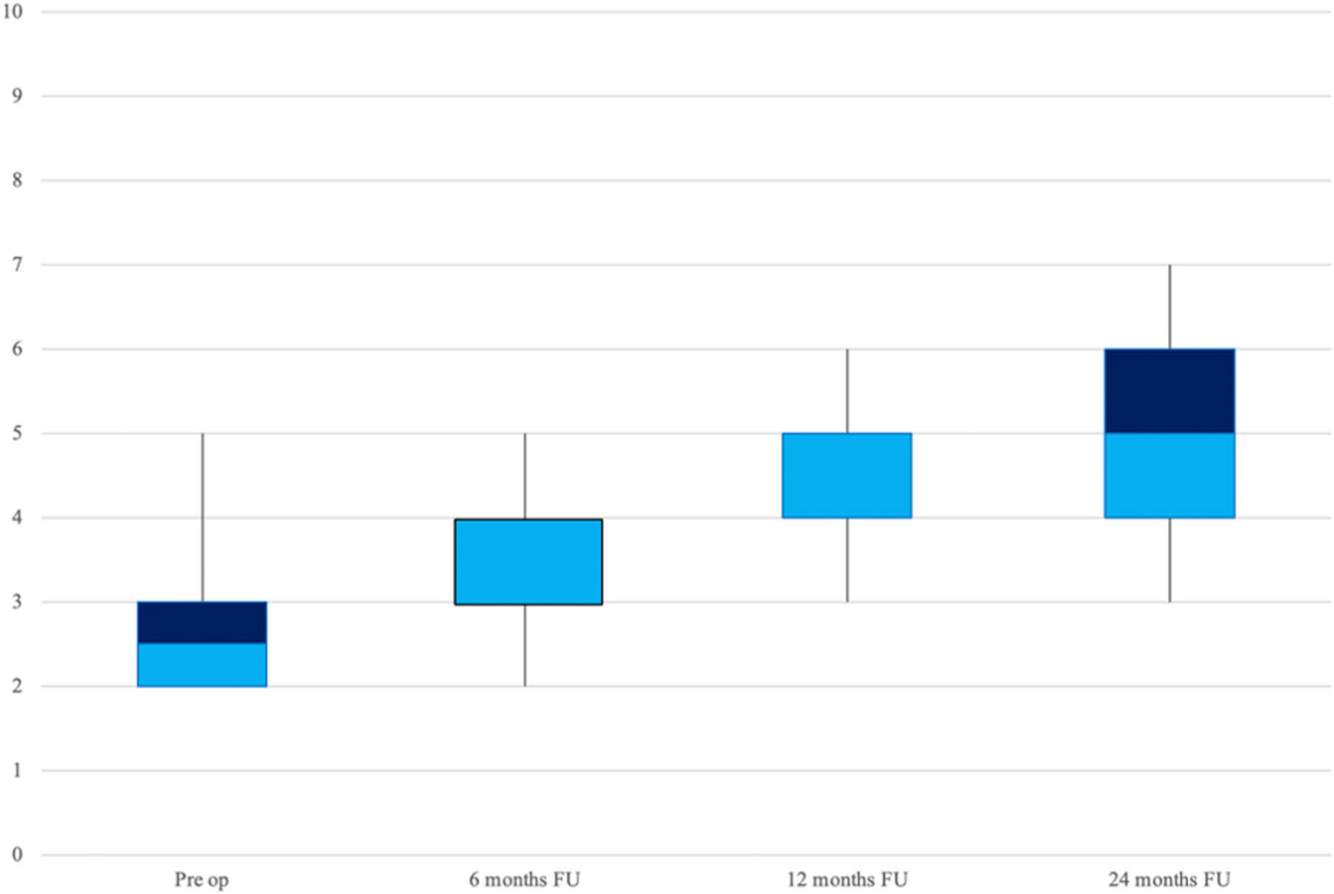
Tegner scores at different FU. Vertical lines represent standard deviations, while squares are statistically significant (*p* < 0.05) across interquantile ranges. FU, follow‐up.

Both IKDC and Tegner scores showed improved values from preoperative through all follow‐ups, as shown in the figures below (Figures 2 and 3). Moreover, patients exhibited a pre‐operative mean IKDC score of 51.48 ± 10.7 prior to surgery, which improved to 77.53 (±13.5) at 6 months FU, 84.5 (±6.2) at 12 months FU and 84.54 (±14.6) at 24 months FU. This clinical improvement was found to be statistically significant (*p* value < 0.05). A change in PROMs that is considered clinically and statistically significant does not always reflect a symptom level that patients perceive as satisfactory, referred to as the patient acceptable symptom state (PASS). Therefore, the number of patients who achieved the PASS was assessed. The threshold value for this analysis was considered to be 75.9 points, as expressed in the paper by Muller et al. [11] in 2016. Regarding our case series, 45 patients (94%) reached the IKDC PASS. Patients with ACL graft failure were all included among those who did not reach the PASS threshold. For the Tegner scores, the median score at preoperative status was 2.5 [2–5], at 6 months FU the score was 4 [2–5], at 12 months it was 5 [3–6], and finally at 24 months FU the median score was 5 [3–7].

### Complications and failures

Patients included in the study were characterized by the absence of complications in 44 cases (91.7%). Concerning the remaining four cases, one patient underwent surgery due to screw breakage with granuloma formation, which resolved after screw removal. The other three cases were complicated by screw breakage with cystic bone degeneration, which was treated with cyst and screw removal and bone grafting. All the patients who experienced this type of complication underwent Xenograft fixation using an absorbable screw. No cases of infection, synovitis or inflammatory reaction were documented in our case series. The complicated cases were not characterized by ACL graft failure.

Xenograft failure was documented in two patients (4.1%), thus showing a graft survivorship of 95.9%. These failures were seen in the primary ACLR group and were all associated with the transtibial technique. To summarize, 4 out of 48 patients experienced post‐operative complications, while 2 out of 48 patients (not included in the complication group) were characterized by ACL graft failure.

## DISCUSSION

The most important findings of the present study are that ACLR with a decellularized porcine xenograft was associated with low complication and failure rates at a mean follow‐up of 20 months, along with significant improvements in PROMs. These positive clinical outcomes are particularly encouraging given the historical concerns surrounding the use of xenografts, which in the 1980s and 1990s were often associated with synovitis and early graft failure, largely due to the use of non‐decellularized tissue. Similarly, a recent study [20] reported a high incidence of post‐operative infections in patients undergoing ACLR with a porcine‐derived xenograft, with 6 out of 29 cases (20.6%) affected. The infections were attributed to contamination by a water‐based pathogen during the graft processing phase, and the authors emphasized the need to improve harvesting and processing protocols before considering widespread use of xenografts. In contrast, in the present series, no cases of post‐operative infection occurred, suggesting that careful graft sourcing and processing mitigate this risk. Moreover, the absence of inflammatory complications suggests that the decellularization process could be crucial to remove major antigens, as expressed in a study by Hunt et al. [5] in 2025, in which no immunologic reactions and outcomes comparable to allograft/autograft were reported.

Another crucial aspect concerning xenograft use in ACLR is graft survivorship and failure rates. Historical studies reported discouraging results: Good et al. [3] described a series of 13 patients treated with a bovine xenograft, with a 77% revision rate due to substantial graft problems or rupture, while van Steensel et al. [16] reported reoperation rates approaching 50% within 12–20 months. In contrast, in our series the reoperation rate for graft rupture was only in 4.1% (2 cases out of 48), which is notably lower than what has been reported in early literature, thus underlining the importance of correct graft processing and patient selection, as shown in a study by Stone et al. [17] where about half the grafts failed (some due to trauma), but the survivors had stable knees with well‐integrated grafts even at 2 decades post‐operatively. Moreover, graft survivorship data showed that xenograft use in middle‐aged patients was associated with comparable outcomes with respect to the use of autograft and allografts in the same population over a short‐term follow‐up.

More encouraging outcomes have been documented in a recent case series, in which Zaffagnini et al. investigated ACLR with a porcine xenograft (Z‐Lig® device) and, consistent with our results, reported significant improvements in IKDC and Tegner scores, along with an absence of clinically relevant immunological reactions, thus supporting the biocompatibility of highly processed porcine tissue.

Similarly, a recent randomized controlled trial including 66 patients (32 allografts and 34 xenografts) with a minimum follow‐up of 24 months reported infections in some xenograft patients due to graft contamination during processing, which negatively affected outcomes in the intention‐to‐treat analysis. However, once patients with infections were excluded, according to the per‐protocol analysis, the xenograft group showed clinical and functional outcomes comparable to the allograft group, with no significant differences in knee stability or PROMs. The significantly high PASS rate (94%) of our case series indicates a high level of patient satisfaction, likely reflecting substantial improvement in pain and functional ability following surgery. This outcome may also be influenced by the demographic profile of the cohort, with an average age of approximately 40 years, as individuals with moderate activity demands may find it easier to reach PASS criteria compared to younger, more athletically active patients.

In our cohort, reoperation was required in four patients (8.3%) due to tibial tunnel enlargement or hardware‐related complications. Tibial tunnel widening has also been documented in other studies investigating xenograft use in ACLR, with some evidence suggesting a slight increase compared to other graft types [20]. However, in our series, complications related to tunnel enlargement occurred primarily in patients who received tibial fixation with bioabsorbable interference screws. Based on these outcomes, we recommend caution in combining xenografts with bioabsorbable tibial fixation devices, as this may increase the risk of tunnel‐related problems.

Finally, the failure rate observed in our study, which was 4.1%, aligns closely with data from a large retrospective cohort study of allograft ACLRs [8]. In that study, out of 215 patients who received allografts, 9 experienced graft failure at short‐term follow‐up, corresponding to a failure rate of approximately 4.2%. This similarity in short‐term failure rates is notable, considering differences in patient populations, surgical techniques, and graft types. It suggests that our results are consistent with larger clinical experiences in the field, reinforcing the reliability of xenograft and allograft options in ACLR with respect to early graft survivorship. Therefore, the clinical relevance of these data lies in the fact that xenograft use could represent a concrete option for ACLR as an alternative to autografts and allografts in middle‐aged patients with not excessively high functional demands; still, appropriate patient selection and optimal graft processing are essential for satisfactory postsurgical outcomes.

One of the main strengths of this study is its multicenter design, involving multiple high‐volume orthopaedic centres where experienced knee surgeons applied the same standardized technique. This setting enhances both the reliability and external validity of the results. Another strength is the relatively large cohort of 48 patients, which exceeds the sample sizes reported in most previous studies on ACL xenografts and provides greater statistical power. The balanced sex distribution further reduces potential gender bias. Finally, the prospective design, with predefined inclusion and exclusion criteria and standardized follow‐up assessments, ensured methodological consistency and limited confounding factors.

Nevertheless, the study has certain limitations that must be acknowledged. One of the most notable limitations is the absence of objective physical performance testing in the post‐operative follow‐up. Quantitative evaluations such as instrumented laxity testing (e.g., KT‐1000 arthrometer measurements), strength assessments, or hop tests would have provided valuable data regarding functional knee stability and recovery. Additionally, a major limitation of the study was the fact that it did not include routine post‐operative imaging, such as magnetic resonance imaging (MRI), to monitor graft postsurgical status. Moreover, the relatively short follow‐up period (mean 20 months) limits the ability to draw definitive conclusions about long‐term graft durability and functional outcomes.

Furthermore, a significant methodological limitation is the lack of randomization and the absence of a control or comparison group. The inclusion of a control group would have allowed for a direct comparison of outcomes and better contextualization of the graft's performance. As such, while our study offers valuable early insights into the clinical feasibility and short‐ to mid‐term efficacy of porcine xenografts in ACLR, the results should be validated through larger, controlled trials in the future. Nevertheless, our empirical data support the use of porcine xenografts in selected patients (i.e., middle‐aged with moderate sport demands), but long‐term studies (e.g., randomized controlled studies in younger and more active individuals, possibly with routine MRI assessment) are essential to define their definitive role.

## CONCLUSION

ACLR using a porcine‐derived xenograft appears to be a safe procedure with a low rate of complications and graft failure. Early outcomes are encouraging, suggesting that this graft could represent a valuable alternative in selected cases. However, further studies with longer follow‐up and larger cohorts are warranted to more comprehensively evaluate the long‐term performance, durability, and functional outcomes of this graft.

## AUTHOR CONTRIBUTIONS

Claudio Zorzi, Arcangelo Russo, Stefano Zaffagnini and Daniele Screpis performed surgeries on the patients included in the study. Stefano Zaffagnini had the original idea and led the article preparation. Francesco Aparo and Gian Andrea Lucidi wrote the first draft of the manuscript. Nicolò Maitan, Giuseppe Gianluca Costa, Simone Natali and Daniele Screpis evaluated the patients at follow‐up. Nicolò Maitan, Giuseppe Gianluca Costa, Simone Natali, Daniele Screpis, Claudio Zorzi and Arcangelo Russo performed a revision of the first draft of the manuscript. All the authors contributed to the final editing of the manuscript, and all the authors read and approved the final version of the manuscript.

## FUNDING

The authors have no funding to report.

## CONFLICT OF INTEREST STATEMENT

Stefano Zaffagnini has received institutional support from Fidia Farmaceutici, Cartiheal, IGEA Clinical Biophysics, Biomet and Kensey Nash; grant support from I+; and royalties from Springer outside the submitted work. The remaining authors declare no conflicts of interest.

## ETHICS STATEMENT

The study protocol was approved by the Institutional Review Board of Istituto Ortopedico Rizzoli (Protocol no. 0012799). All the patients signed a written informed consent prior to inclusion in the study.

## Data Availability

All the data are available upon request from the corresponding author.
